# How does the brain process rhythm?

**DOI:** 10.7554/eLife.02658

**Published:** 2014-03-25

**Authors:** Elizabeth Kirkham

**Affiliations:** Department of Psychology, University of Sheffield, Sheffield, United Kingdomekirkham1@sheffield.ac.uk

**Keywords:** science writing competition, outreach, open access

## Abstract

A region of the brain called the putamen has a central role in our ability to keep a beat in our head.

This article by Elizabeth Kirkham (pictured) was the winning entry in the Access to Understanding science-writing competition for PhD students and early career post-doctoral researchers organized by Europe PubMed Central in partnership with The British Library. Entrants were asked to explain to a non-scientific audience, in less than 800 words, the research reported in a scientific article and why it mattered.

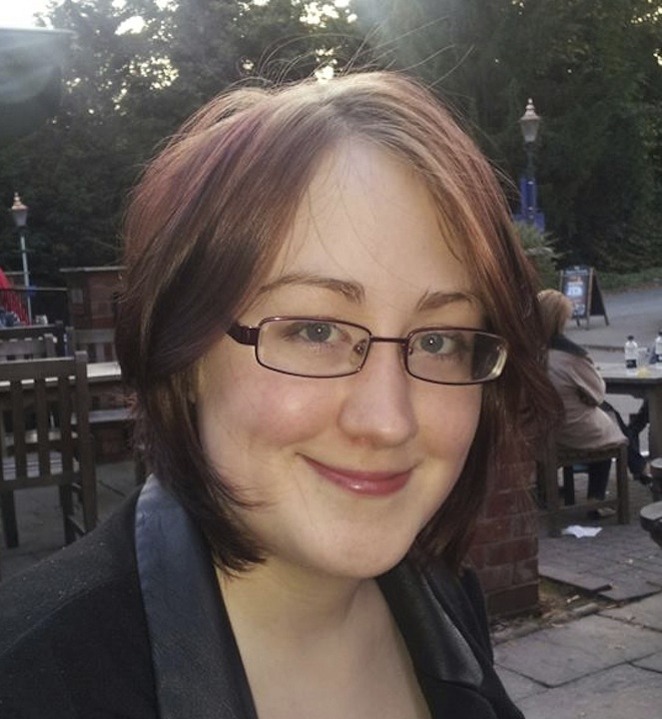

Most people have little trouble recognising and following the beat in a piece of music. We can even continue to play the beat in our minds once a song has finished. However, few of us are aware that our knack for holding a beat in our head actually makes life easier. The capacity to identify patterns in streams of sound supports many forms of human behaviour, including moving, speaking and listening.

If the ability to generate this internal rhythm is disrupted, such as in Parkinson’s disease, problems begin to arise. People with Parkinson’s disease have difficulty with psychological tasks, such as holding a beat in their head, as well as with practical tasks, such as walking. The more we know about the regions of the brain that are causing these difficulties, the more effective we will be in designing treatments to combat them. Previous research suggests that a set of brain structures known as the basal ganglia have a role in identifying and following a beat.

The more we know about the regions of the brain that are causing these difficulties, the more effective we will be in designing treatments to combat them.

The basal ganglia are a network of brain regions that are involved in movement and action. Many of the regions within the basal ganglia appear to play a role in the processing of rhythm. One such region is the putamen, a round structure near the centre of the brain. Studies measuring levels of brain activity have found that the putamen is active when a person is listening to a beat. However, it is not clear whether the putamen is merely identifying the presence of the beat, or whether it is actually helping us to recreate that beat in our minds.

Jessica Grahn of the University of Western Ontario and James Rowe of Cambridge University and the MRC Cognition and Brain Sciences Unit, also in Cambridge, designed a study that allowed them to distinguish between these two possibilities (Grahn and Rowe, 2013). They began by creating small snippets of sound. Some of these sound-bites contained a beat, while others did not. Then they combined pairs of sound-bites to produce four different types of sequences: in the first sequence a sound-bite without a beat was followed by a different sound-bite without a beat; in the second a sound-bite without a beat was followed by one with a beat; in the third a sound-bite with a beat was followed by a one with a version of the same beat; and in the fourth sequence a sound-bite with a beat was followed by a sound-bite with a faster or slower version of the same beat.

Grahn and Rowe asked the participants in their study to listen to the different sequences whilst a scanner measured how their brain responded. In order to measure this brain activity, they used functional magnetic resonance imaging (fMRI). This is a form of brain imaging that allows us to see which regions of the brain are involved in a particular task: it does this by measuring the amount of oxygen that a specific region of the brain is using relative to other regions. If a brain region is using a lot of oxygen during a task, it is assumed that it is involved in carrying out the task.

Grahn and Rowe found that the putamen responded differently to different beat sequences. When there was no beat, the putamen was not active. Similarly, when participants heard a new beat, the putamen did not respond. By contrast, when participants heard the same beat twice, the putamen was highly active. It was also active, but to a lesser extent, when the sequence involved the same beat played at different speeds.

These results suggest that the putamen was not responding to the presence of a beat per se, but was processing the continuation of the beat across the sequence. This supports the theory that the putamen is involved in our ability to recreate a beat in our head. Prior to this study, researchers knew that the putamen was involved in beat processing, but they did not know what its specific role was. The work of Grahn and Rowe shows that the putamen is important for the mental generation of a beat.

In addition to advancing our knowledge of the putamen’s role in beat processing, these findings could have clinical implications. People with Parkinson’s disease are capable of identifying a beat in a piece of music, but have difficulty when it comes to reproducing the beat in their own minds. This study shows that this pattern of symptoms could be caused by damage to the putamen, thus highlighting the need to consider this part of the brain as a target for future treatment of Parkinson’s disease.

## References

[bib1] Grahn JA, Rowe JB (2013). Finding and feeling the musical beat: striatal dissociations between
detection and prediction of regularity. Cerebral Cortex.

